# On the Therapeutical Action of Koch's Remedy

**Published:** 1891-03-21

**Authors:** 


### THE PRACTITIONER'S MIRROR AND RETROSPECT.

[*The Editor will be glad to receive offers of co-operation and contributions from members of the profession. All letters should be addressed to* THE EDITOR, THE LODGE, PORCHESTER SQUARE, LONDON, W.]

### MEDICINE.

### ON THE THERAPEUTICAL ACTION OF KOCH'S REMEDY.

How does the remedy act in overcoming tubercular disease? In a paper recently read before the Bristol Medico-Chirurgical Society, Dr. Watson Williams remarked that in this connection the writings of John Hunter on the specific action of mercury in syphilis seem most interesting and much to the point. Hunter proved that the virus of syphilis was of the nature of a ferment (without, of course, recognising the micro-organism which we now believe to be essential to this specific ferment), just as we now recognise that the pathological action of tuberculosis is due to the ferment produced by the tubercle bacilli in the tissues.

Speaking of the operation of mercury on the syphilitic virus, he says: "Mercury may be supposed to act in three different ways in curing venereal disease. First, it may unite with the poison chemically and decompose it, by which means its powers of irritation may be destroyed; secondly, it may carry it out of the constitution by evacuation; or, thirdly, it may produce an irritation in the constitution which counteracts the venereal and entirely destroys it;" and it is in one of these possible modes of action that Koch's lymph must operate.

It has been suggested that Koch's lymph acts by causing necrosis of the tubercular tissue, which is thus bodily thrown off, or that the bacilli are carried out in the evacuations of lymph, as seen on the surface of lupus deposits. But many of the most remarkable cases of improvement have been those in which the local reaction has been almost nil, in which the tubercular deposits disappear without necrosis and without any obvious exudation of lymph.

Hunter further stated that mercury did not act by uniting chemically with the poison of syphilis, and in the same manner we can prove that the lymph does not act by uniting chemically with the tubercular virus. If this was the case, the beneficial results would be in proportion to the dose administered, and the reaction in proportion to the amount of tubercular tissue present. But just as Hunter proved that mercury must act by stimulating the syphilitic tissues, so must we fall back on the hypothesis that the therapeutical effects of bacteriological products are due to their acting as stimulants or slight irritants of the tissue of the cells, so stimulating them to greater activity and increasing their power of resistance, and at the same time exerting a deleterious influence on the special bacteria that produces them.

There is soon acquired a remarkable tolerance of the poison of the lymph. It is certainly very important to bear in mind that the degree of reaction to the lymph varies according to the individual susceptibility of the patient rather than according to the amount of tubercular deposit present.

But to explain the therapeutical action of the drug by the simple statement that an acquired tolerance is induced is to beg the question.

"At the present time we know that all unicellular organisms possess the power of selecting their food, of taking up the useful, and rejecting the useless substances, so that one can hardly avoid the conclusion that the acts of these monads are those of conscious beings."

"If the power of selecting food is possessed by the structureless mass of protoplasm," continues Bunge, "why should it not be also a function of the intestine, and, for instance, the epithelial cells of an intestine to select the fat drops in our food, and reject the pigment granules. We know that the epithelium of our intestines prevents the absorption of a whole series of poisons, in spite of the fact that the latter are easily soluble in the gastric and intestinal juices."

The intestinal wall is covered with epithelium, and every epithelial cell is in itself an organism, a living being, so to speak, with the most complex functions; it takes up food by the active contraction of its protoplasm in the same way as observed in independent naked animal cells, such as amœbæ and rhizopods.

From his experiments Armand Ruffer has concluded that in the Peyer's patches of the intestine of a rabbit some of the cellular elements, the lymphocytes, wander to the surface of the intestine, seize on the micro-organisms present in that situation, and carry them to the interior.

Now, wherever tubercular deposits occur there are present giant cells, and Armand Ruffer's observations on the action of lymphocytes and giant cells, or macrophages, lead him to affirm that inasmuch as the tubercle nodule consists of a number of extremely active amœboid cells, which all have the power of seizing, destroying, and digesting tubercle bacilli, he is of opinion that the processes taking place in the tubercle nodule, as long as the anatomical elements composing it are alive, serve a useful purpose.

If we are right in believing that Koch's remedy causes tubercular deposits to disappear by stimulating the giant cells and lymphocytes to increased activity, we have an explanation of Koch's observation that it acts on living tuberculous tissue alone, having no effect on dead tuberculous tissue. Thus, too, we may explain the deplorable fact that in advanced cases of phthisis the remedy has little or no effect, for when the vitality of the tissues has become exhausted it is no longer possible to stimulate their elements to renewed attacks on the invading bacilli. In some of the most advanced cases of phthisis, with crowds of bacilli in the sputum, the reaction is least marked. In confirmation of this view, we would call to mind the appearances of the bacilli after the inoculations, very closely resembling the altered appearances of some of the bacilli seen by Ruffer lying semi-digested in the interior of the giant cells.

The latest communication of Metschnikoff seems to support these views as to the mode of action of the Koch remedy: "Years ago it was established in M. Pasteur's laboratory that the refractory organism, *i.e.*, the organism which is naturally insusceptible to a microbe or which has been rendered so by vaccination, instead of being an unfavourable soil for the preservation of virulence, tends to reinforce the property." Thus the virulence of the chicken cholera microbe is greatly increased by inoculation into a vaccinated cock.

Further, it has been shown by the experiments of Charrin, Gamaleia, and Selander, that in three microbial diseases remarkable for their pronounced toxic character, viz., vibrionic septicæmia, pyocyanic disease, and hog cholera affecting the rabbit, when the rabbit has been rendered immune from either of these by previous vaccination, and is then inoculated, that the immunity is due to the lymphocytes attacking and destroying the microbes, thus preventing their forming toxines; and that the immunity is not due to their becoming tolerant of the toxine is shown by the fact that they are just as susceptible to the poisonous action of these toxines as the unvaccinated animal is. It would appear, then, that in these affections, at any rate, the immunity is conferred by the lymphocytes and microphages being taught to attack and overcome the invading micro-organism, and in the same way we may suppose that the lymph when inoculated stimulates the defensive cells of tubercular tissue to resist tho tubercle bacillus, and by preventing the further formation of the tubercular toxines, tends to stop the night sweats and to improve the general condition.

The practical point that such therapeutical views suggest is that as good, if not better, results will be obtained by the administration of the smaller and safer doses than with the comparatively larger dose which has already led to disastrous results, which could not have been foreseen; and further, that inasmuch as in acquired specific diseases the infecting microbe is overcome by complex biological processes, it behoves us to be extremely cautious not to interfere with these natural therapeutics of the tissues. That powerful chemical antiseptic or germicidal remedies having lamentably failed in overcoming tubercular phthisis, their employment as bactericides is therefore contra-indicated, and that in tuberculosis, as in enteric fever and other like affections, it is better to help the patient to kill his own germs than to attempt their destruction by intestinal antisepsis, antiseptic inhalations, hot air, cold air, and a hundred and one other things equally useless and often harmful.

